# An appraisal of analytical tools used in predicting clinical outcomes following radiation therapy treatment of men with prostate cancer: a systematic review

**DOI:** 10.1186/s13014-017-0786-z

**Published:** 2017-03-21

**Authors:** Elspeth Raymond, Michael E. O’Callaghan, Jared Campbell, Andrew D. Vincent, Kerri Beckmann, David Roder, Sue Evans, John McNeil, Jeremy Millar, John Zalcberg, Martin Borg, Kim Moretti

**Affiliations:** 1 South Australian Prostate Cancer Clinical Outcomes Collaborative (SA-PCCOC), Adelaide, Australia; 2 Freemasons Foundation Centre for Men’s Health, University of Adelaide, Adelaide, Australia; 3 Centre for Population Health Research, University of South Australia, Adelaide, Australia; 4 Epidemiology & Preventative Medicine, Monash University, Clayton, Australia; 5 Radiation Oncology, Alfred Health, Melbourne, Australia; 6 Adelaide Radiotherapy Centre, Adelaide, Australia; 7 SA Health, Repatriation General Hospital, Urology Unit, Daws Road, Daw Park, 5041 SA Australia; 8 Flinders Centre for Innovation in Cancer, Bedford Park, Australia; 9 Joanna Briggs Institute, University of Adelaide, Adelaide, Australia; 10 Discipline of Surgery, University of Adelaide, Adelaide, Australia; 11 School of Public Health and Preventive Medicine, Monash University, Clayton, Australia

**Keywords:** Prostate cancer, Systematic literature review, Nomogram, Outcomes, Survival, Biochemical recurrence

## Abstract

**Background:**

Prostate cancer can be treated with several different modalities, including radiation treatment. Various prognostic tools have been developed to aid decision making by providing estimates of the probability of different outcomes. Such tools have been demonstrated to have better prognostic accuracy than clinical judgment alone.

**Methods:**

A systematic review was undertaken to identify papers relating to the prediction of clinical outcomes (biochemical failure, metastasis, survival) in patients with prostate cancer who received radiation treatment, with the particular aim of identifying whether published tools are adequately developed, validated, and provide accurate predictions. PubMed and EMBASE were searched from July 2007. Title and abstract screening, full text review, and critical appraisal were conducted by two reviewers. A review protocol was published in advance of commencing literature searches.

**Results:**

The search strategy resulted in 165 potential articles, of which 72 were selected for full text review and 47 ultimately included. These papers described 66 models which were newly developed and 31 which were external validations of already published predictive tools. The included studies represented a total of 60,457 patients, recruited between 1984 and 2009. Sixty five percent of models were not externally validated, 57% did not report accuracy and 31% included variables which are not readily accessible in existing datasets. Most models (72, 74%) related to external beam radiation therapy with the remainder relating to brachytherapy (alone or in combination with external beam radiation therapy).

**Conclusions:**

A large number of prognostic models (97) have been described in the recent literature, representing a rapid increase since previous reviews (17 papers, 1966–2007). Most models described were not validated and a third utilised variables which are not readily accessible in existing data collections. Where validation had occurred, it was often limited to data taken from single institutes in the US. While validated and accurate models are available to predict prostate cancer specific mortality following external beam radiation therapy, there is a scarcity of such tools relating to brachytherapy. This review provides an accessible catalogue of predictive tools for current use and which should be prioritised for future validation.

**Electronic supplementary material:**

The online version of this article (doi:10.1186/s13014-017-0786-z) contains supplementary material, which is available to authorized users.

## Background

### Rationale

Prostate cancer is the most prevalent cancer in men globally, with 1.4 million new cases reported in 2013 [1]. Prostate cancer cases increased by 217% between 1990 and 2013 as a result of population growth and aging and increased uptake of opportunistic screening, particularly in developing countries [1]. Prostate cancer remains the leading cause of death among males in 24 of 188 countries covered by the Global Burden of Disease Cancer Collaboration [1].

Prostate cancer treatments are varied and include: deferred treatment (active surveillance), watchful waiting, radical prostatectomy, radiation therapy (with or without androgen deprivation therapy) or androgen deprivation therapy (ADT) [2, 3]. Each treatment will achieve different outcomes in terms of oncology (e.g., survival or time to biochemical recurrence), adverse events and patient reported outcomes such as urinary incontinence and impotence. These outcomes are important considerations when selecting a treatment for prostate cancer patients and are considered in the context of patient age, life expectancy, co-morbidities, tumour size, grade and stage and other risk indicators that influence outcomes and treatment choice. Determining which treatment choice is optimal for each patient remains an important challenge, particularly where directly relevant randomised controlled data is lacking.

To aid this decision making process, a number of tools have been developed with nomograms and risk stratification systems most commonly used [4]. Nomograms are graphic tools developed to aid clinical decision making and are well established in clinical practice for prostate cancer, particularly for assisting selection of treatment approaches based on risk stratification. Such tools have been shown to improve prediction of outcomes when compared with clinician judgement alone [5, 6]. Unfortunately most nomograms currently in use are likely to be based on dated treatment modalities. Furthermore predictions based on observations made in one setting may not be accurate in another (e.g., where ethnicity or health services differ). Extrapolation of published international results to local practice is a known pitfall that has potential to mislead both clinicians and patients [7]. These limitations are particularly relevant to predictive tools designed for use in patients treated with radiation therapy as this modality has changed significantly over the past decade.

### Objectives

We aim to identify papers predicting clinical outcomes for patients with prostate cancer who have been treated with radiation therapy. We particularly set out to assess if the tools identified were adequately developed, validated and provide accurate predictions.

## Methods

### Protocol and registration

A systematic literature review protocol was developed for this study and registered before searches commenced with PROSPERO, an international prospective register of systematic reviews. The protocol can be accessed at: http://www.crd.york.ac.uk/PROSPERO/display_record.asp?ID=CRD42015025428.

### Inclusion criteria

Papers were eligible for inclusion where they met the following criteria; Population: Patients with prostate cancer. Exposure: Treatment with radiation therapy (including external beam radiation therapy and/or brachytherapy). Outcome: The generation or validation of a tool for the prediction of clinical outcomes (biochemical failure [BF], progression to metastases, prostate cancer specific survival, overall survival). Papers had to be written in English and published post July 2007. This date was chosen as it is the search date up to which a previous systematic review of prognostic tools for prostate cancer treated by any therapy was undertaken [4]. Studies were included which described tools using variables which are currently available in a clinical setting. This excluded papers including genetic or molecular variables.

### Information sources

Searches were conducted of the Medline database (PubMed interface) and the EMBASE database.

### Search

Disease-specific search terms included: prostate cancer, prostatic neoplasms, cancer of the prostate, adenocarcinoma of the prostate, prostatic cancer, prostate gland cancer and prostate tumour. Treatment specific search terms included: radiation therapy, radiotherapy, external beam radiotherapy, EBRT, brachytherapy, high dose radiotherapy, low dose radiotherapy and targeted radiotherapies. Outcome-specific search terms included: overall survival, progression-free survival, PFS, mortality, event free survival, EFS, disease free survival, prostate cancer specific survival, progression to metastases, time to progression, TTP, biochemical recurrence, BCR, biochemical failure, neoplasm recurrence. Search terms used to identify predictive models included: predictive tools, nomograms, risk stratification, Partin tables, regression tree analysis, Artificial Neural Networks, CAPRA-S or CAPRA score, risk estimates, algorithms, predictive accuracy, diagnostic test accuracy, Kattan tables/nomograms.

### Study selection

Study selection included three phases. The titles and abstracts of all studies identified by the search strategy were compared to the inclusion criteria detailed above by two authors working independently (ER and MOC). All studies that appeared likely to meet the inclusion criteria were progressed to full-text review. All discrepancies, where authors reached different conclusions about the same papers, were resolved through discussion. The full-texts of these papers were then retrieved and assessed against the inclusion criteria, again by two authors (ER, JC or MOC) working independently in order to minimise the impact of human error. Studies that were identified as meeting all inclusion criteria were included in the review, while those which did not were excluded. Again, where there were differences in the authors’ conclusions consensus on the correct decision was reached through discussion. Finally, the reference lists of included papers were screened for any additional relevant papers which may have been missed by the search strategy. All new titles identified were then reviewed as described above.

### Data collection process and data items

After full text review, data extraction was undertaken by one reviewer (ER, JC or MOC). Items for extraction included: manuscript identifiers (author, contact, country, setting), study methods, population studied (inclusion criteria, exclusion criteria, baseline characteristics – dates of recruitment, age, ethnicity, number of patients, primary treatment, treatment subtype, adjuvant therapies, neoadjuvant therapies), and predictive model characteristics (type of model, variables included, if internal validation was reported and the type, external validation, variable definitions, if variables were readily available, sample size, number of events, definition of outcome, model accuracy, sensitivity, specificity, concordance index and receiver operator curve area under the curve). For assessment as to whether or not variables were considered ‘readily available’ the minimum data set used by the only national prostate cancer registry (Prostate Cancer Outcomes Registry, Australia and New Zealand Australian [8]) was used as a guide.

### Quality assessment

Quality assessment was performed by two reviewers (ER, JC or MOC) for each paper. Four questions were selected for this assessment: 1. Was the defined representative sample of patients assembled at a common (usually early) point in the course of their disease? 2. Was patient follow-up sufficiently long and complete? 3. Were outcome criteria either objective or applied in a ‘blind’ fashion? And 4. If subgroups with different prognoses were identified, did adjustment for important prognostic factors take place? These questions were selected from the Centre for Evidence Based Medicine ‘Critical appraisal of prognostic studies’ tool [9]. Discrepancies between reviewers were discussed and consensus reached. Questions that were answered positively >75% of the time were considered to present a low risk of bias, those ≤75 to >50% a moderate risk of bias, and any ≤50% a high risk of bias. Data extraction and quality assessment were performed using the online tool ‘Covidence’.

## Results

**Fig. 1:**
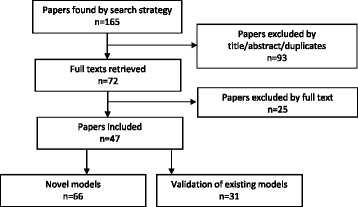
Flow diagram

The search strategy resulted in 165 potentially relevant abstracts/articles and these were reduced to 72 once duplicates were removed and title and abstracts were screened (Fig. 1). The full-text of these papers was reviewed against the inclusion criteria (reasons for exclusion are reported in Additional file 1: Table S1a and b) and 47 finally selected. Study recruitment periods varied considerably with the earliest patients being from 1984 [10] and the latest 2009 [10–13] (Table 1). The populations of individual studies varied from 80 [14] to 7,839 [14, 15] with a combined population of 60,457 (Tables 2, 3 and 4). The majority of studies were retrospective (*n* = 38), however seven studies recruited prospective cohorts (for one study [16] it was not stated whether it was retrospective or prospective).

**Table 1:** Summary of papers describing prognostic tools relating to clinical outcomes following radiation therapy (2007–2015)

Author	Recruitment window	Country	Population	Outcome	Study type	Setting
Bittner [27]	1995–2006	USA	Prostate cancer patients treated with brachytherapy	BFFF, PCSM	Retrospective	Single centre
Buyyounouski [38]	1989–2000	Canada, Aust, USA	Men previously treated with EBRT for clinically localized prostate adenocarcinoma and subsequently diagnosed with BCF.	PCSM	Retrospective	Multi-centre
Cooperberg [39]	1995–2007	USA	Men enrolled in CaPSURE	PCSM	Retrospective	Multi-centre (CaPSURE Registry)
Cooperberg [40]	1995–2008	USA	Men with localized disease who underwent prostatectomy, received external-beam radiation, or received primary androgen deprivation; and had at least 6 months of follow-up recorded.	10 year PCSM	Retrospective	Multi-centre (CaPSURE Registry)
D’Ambrosio [41]	1989–2004	USA	Men with prostate cancer treated with RT.	BCF	Retrospective	Single centre
D’Amico [42]	1991–2005	USA	Men with high-risk prostate cancer (locally or advanced) and 10 year life expectancy treated with brachytherapy who were observed for a min of 2 years.	PCSM and presence of hormone-refractory metastatic prostate cancer.	Prospective	Multi-centre
D’Amico [43]	1988–2004	USA	Men who underwent RT for prostate cancer for at least 1 high-risk feature.	PCSM	Prospective	Multi-centre
Delouya [19]	2002-Not stated	Canada	Men with low or intermediate-risk prostate cancer treated with brachytherapy, EBRT within a phase II or III research protocol, or ERBT outside of a protocol.	BCF	Retrospective	Single centre
Denham [44]	1996–2000	Australia & New Zealand	Men with locally advanced prostate cancer receiving RT	PCSM	Prospective	Multi-centre
Engineer [9]	1984–2004	India	Patients with a histological diagnosis of prostate cancer	BFFF, PCSM, DM, BCF, OS	Retrospective	Single centre
Feng [28]	1998–2008	USA	Men with clinically localized prostate cancer treated with EBRT.	FFM, PCSM, BFFF, OS	Retrospective	Single centre
Frank [45]	1996–2006	USA, Canada, Netherlands.	Men with prostate cancer treated with brachytherapy with at least 30 months of follow-up.	PSA failure.	Retrospective	Multi -centre
Frank [25]	1998–2006	USA	Men with prostate cancer treated with permament 125 I brachytherapy.	5 year BFFF	Retrospective	Single centre
Halverson [46]	1998–2008	USA	Men with clinically localized prostate cancer treated with EBRT with or without adjuvant ADT	BFFF	Retrospective	Single centre
Huang [47]	1993–2003	USA, Australia	Men with clinical Stage T1c-T3N0M0 prostate adenocarcinoma treated with EBRT with or without a high-dose rate brachytherapy boost.	BCF, DM, PCSM,OS.	Retrospective	Single centre
Kaplan [12]	2000–2009	Israel	Patients with prostate cancer treated with 125 I- brachytherapy.	BFFF	Retrospective	Single centre
Krishnan [20]	2003–2008	Canada	Men with intermediate-risk prostate cancer with a minimum follow-up of 3 years.	BCF	Retrospective	Single centre
Kubicek [48]	1998–2004	USA	Men with biopsy proven T1-T2 prostate adenocarcinoma treated with EBRT & LDR.	CSS	Retrospective	Single centre
Marshall [11]	1990–2009	USA	Men treated with brachytherapy for biopsy-proven prostate adenocarcinoma.	BCF	Retrospective	Single centre
McKenna [49]	1998–2003	USA	Men with biopsy-proved prostate cancer who had MRI imaging prior to EBRT.	Metastatic recurrence and BCF	Retrospective	Single centre
Murgic [50]	1998–2008	USA	Men with clinically localized prostate adenocarcinoma treated with EBRT.	BFFF, FFM,PCSM and OS	Retrospective	Single centre
Potters [16]	Not stated	USA	Prostate cancer patients treated with brachytherapy.	9-year BFFF	Retrospective	Multi-centre
Proust-Lima [51]	Not stated	USA	Men treated for localized prostate cancer with EBRT.	BCF	Prospective	Multi-centre
Qian [52]	1998–2008	USA	Men who were treated with EBRT for clinically localized prostate cancer with or without neoadjuvant or adjuvant ADT.	BFFF, FFM,OS, PCSM.	Retrospective	Single centre
Rodrigues [14]	Not stated	Canada	Men with prostate cancer.	BFFF, OS	Retrospective	Multi-centre (GUROC ProCaRS database)
Sabolch [53]	1998–2008	USA	Men treated for localized prostate cancer with EBRT.	BFFF, FFM,OS, PCSM.	Prospective	Single centre
Sanpaolo [21]	2000–2004	Italy	Men with T1-T3 NO prostate cancer.	BCF	Retrospective	Single centre
Slater [54]	1991–1999	USA	Randomly selected prostate cancer patients treated with proton and photon beam therapy.	bNED	Retrospective	Single centre
Spratt [55]	1997–2008	USA	Men with localized prostate cancer were treated with IMRT.	BCF, DMFS, BCR	Retrospective	Single centre
Steigler [56]	1996–2000	Australia & New Zealand	Men with localised advanced prostate cancer treated with RT and experienced BCF prior to clinical failure or secondary theraputic intervention.	TTBF, PCSM,distant progression and STI from BCF	Retrospective	Multi-centre
Sylvester [57]	1988–1992	USA	Men with clinically localized prostate cancer treated with implanted I-125.	15 year BFFF,CSS and OS.	Prospective	Consecutive case series
Taylor [58]	Not stated	USA	Men with localized prostate cancer,NO/MO treated with RT.	Clinical recurrence (local, regional or distant)	Retrospective	Multi-centre
Thames [59]	1987–1995	USA	Men with clinical stages T1b, T1c, and T2 N0M0 biopsy proven prostate adenocarcinoma.	BCF	Retrospective	Multi-centre
Vainshtein [18]	1998–2008	USA	Men with localized prostate cancer treated with EBRT, +/− ADT	FFM, PCSM.	Prospective	Single centre
Vance [60]	1998–2008	USA	Men with clinically localized prostate cancer treated with EBRT, with or without neoadjuvant or adjuvant ADT.	BFFF, DMFS, PCSM & OS.	Retrospective	Single centre
Wattson [61]	1991–2007	USA	Men with high-risk prostate cancer.	PCSM	Retrospective	Multicentre
Westphalen [62]	1998–2007	USA	Prostate cancer patients who underwent endorectal MR and MR spectroscopy prior to EBRT.	BCF	Retrospective	Multi-centre (national administrative data set)
Williams [17]	1991–2002	US, Canada, Australia	Men with clinical T1–4 N0/X M0/X prostate adenocarcinoma treated with EBRT.	BCF	Retrospective	Multi-centre
Yoshida [15]	2003–2008	Japan	Men with histologically-proven prostate adenocarcinoma, treated with HDR-ISBT.	5 year PSA failure and OS	Retrospective	Single centre
Yu [63]	1987–2001	USA	Men with prostate cancer treated with EBRT.	BCF	Retrospective	Single centre
Yu [64]	1993–2002	USA	Men newly diagnosed with clinically node-negative, localized adenocarcinoma of the prostate treated with EBRT.	BCF	Retrospective	Single centre
Zaorsky [65]	1992–2004	USA	Men with clinical stage T1-4, NO/NX-N1, MO adenocarcinoma of the prostate received RT with or without adjuvant ADT.	BCF,DM, OS.	Retrospective	Single centre
Zelefsky [66]	1988–2004	USA	Men with clinically staged T1-T3 node-negative prostate cancer treated with 3D-CRT or IMRT.	DMFS, BFFF.	Retrospective	Single centre
Zelefsky [67]	1998–2000	USA	Men with clinically localized prostate cancer treated with 3D-CRT or IMRT.	DM,PCSM,BFFF	Retrospective	Single centre
Zelefsky [68]	1988–2004	USA	Men with Stage T1-T3 prostate cancer treated with 3D-CRT or IMRT.	PSA relapse	Retrospective	Single centre
Zelefsky [10]	1998–2009	USA	Men with clinically localised prostate cancer treated with brachytherapy.	BFFF	Retrospective	Single centre
Zumsteg [69]	1992–2007	USA	Men with intermediate-risk prostate cancer, but without high-risk features treated with EBRT.	BCF, BFFF, LF,PCSM, DM.	Retrospective	Single centre

*Abbreviations*: *OS* overall survival, *CaPSURE* Cancer of the Prostate Strategic Urologic Research Endeavour, *RT* radiotherapy, *BCF* bio chemical failure, *BFFF* bio chemical freedom from failure, *PCSM* prostate cancer specific mortality, *PSA-RFS* prostate-specific antigen recurrence-free survival, *LF* local failure, *DM* distant metastases, *DMFS* distant metastases-free survival, *FFM* freedom from metastases, *HDR-ISBT* high-dose-rate interstitial brachytherapy, *TTBF* time to bio chemical failure, *STI* secondary therapeutic intervention, *bNED* bio chemical no evidence of diseaese, *2D-CRT* 2D - Conformal radiotherapy, *3D-CRT* 3D -Conformal radiotherapy, *EBRT* external beam radiotherapy, *LDR brachytherapy* low dose rate brachytherapy, *NO/NX* no nodal involvement, *I-125* Iodine 125 brachytherapy

**Table 2:** Prognostic tools relating to brachytherapy

Author	Model type	Variables	Variable readily available?	Validation (I/E)	Accuracy	Metric	Sample size (events)	Outcome	Treatment
**Frank** [25]	Survival (Nomogram presented)	Biopsy gleason score, clinical stage, EBRT, pre-treatment PSA,	Yes	External validation of Prostogram	0.49; 95% CI 0.37–0.61	c-index	208 (15)	5 year BFFF	Brachytherapy
**Kaplan** [12]	Survival (Nomogram presented)	Kattan’s: Pretreatment PSA level, Gleason score, clinical stage, adjuvant EBRT	Yes	External validation of Kattan	0.51	c-index	747 (31)	BFFF	125 iodine brachytherapy
**Frank** [47]	Survival (Nomogram presented)	Pretreatment PSA level, Gleason sum score, T stage, and EBRT	Yes	External validation of Prostogram	0.66	c-index	683 (29)	BCF	Brachytherapy
**Zelefsky** [10]	Proportional hazards regression (Nomogram presented)	Clinical stage, Gleason, pretreatment PSA	Yes	Not stated	0.70	c-index	1466 (NR)	BCF	Brachytherapy
**Potters** [16]	Survival (Cox,Nomogram presented)	Clinical stage, Biopsy Gleason sum, Isotope used, EBRT, D90, pretreatment PSA	No, includes isotope used, D90	Internal (bootstrapping)	0.71	c-index	5931 (NR)	9-year BFFF	Brachytherapy
**D’Amico** [42]	Survival Model (Fine and Gray)	Year of brachytherapy, Log (PSA)per unit increase, Gleason score, Age	Yes	Not stated	Not stated	NA	221 (32)	PCSM and presence of hormone-refractory metastatic prostate cancer	Brachytherapy
**Sylvester** [57]	Survival model (Cox)	PSA only (<10, 10.1–19.9, >20)	Yes	Not stated	Not stated	NA	215 (NR)	15 year BFFF	Brachytherapy
**Sylvester** [57]	Survival model (Cox)	PSA only (<10, 10.1–19.9, >20)	Yes	Not stated	Not stated	NA	215 (NR)	15 year PCSM	Brachytherapy
**Sylvester** [57]	Survival model (Cox)	PSA only (<10, 10.1–19.9, >20)	Yes	Not stated	Not stated	NA	215 (NR)	15 year OS.	Brachytherapy
**Bittner** [27]	Survival model (Cox)	Number of biopsy cores, PSA, Gleason score, % positive biopsies, V100, EBRT, Risk group, hypertension, Tobacco use, perineural invasion	No, tobacco use, V100, hypertension included.	Not stated	Not stated	NA	1613 (NR)	BFFF	Brachytherapy
**Bittner** [27]	Survival model (Cox)	PSA, Gleason score, % positive biopsies, EBRT, Risk group, hypertension	No, hypertension	Not stated	Not stated	NA	1613 (NR)	PCSM	Brachytherapy
**Bittner** [27]	Survival model (Cox)	Number of biopsy cores, age at implant, BMI, V100, D90, EBRT, Risk group, hypertension, diabetes, Tobacco use	No, BMI, V100, D90, hypertension, diabetes included	Not stated	Not stated	NA	1613 (NR)	OS	Brachytherapy
**Cooperberg** [39]	Survival model (Cox)	CAPRA scores (based on PSA, Biopsy Gleason, Age at diagnosis, clinical tumour stage and % biopsy cores positive for cancer)	Yes	Not stated	Not stated	NA	1441 (17)	PCSM	Brachytherapy
**Yoshida** [15]	Survival model	PRIX score derived from PSA, Gleason and clinical stage	Yes	External	Not stated	NA	100 (9)	5 year BCF	HDR-ISBT
**Yoshida** [15]	Survival model	PRIX score derived from PSA, Gleason and clinical stage	Yes	External	Not stated	NA	100 (9)	5 year OS	HDR-ISBT
**Marshall** [11]	Survival model (Cox)	Age, Risk group, hormone treatment, Total BED	Yes	Not stated	Not stated	NA	2495 (251)	BCF	Brachytherapy

*Abbreviations OS* overall survival, *BCF* bio chemical failure, *BFFF* bio chemical freedom from failure, *PCSM* prostate cancer specific mortality, *HDR-ISBT* high-dose-rate interstitial brachytherapy, *EBRT* external beam radiotherapy, *NR* not reported, *NA* not applicable

**Table 3:** Prognostic tools relating to external beam radiation therapy

Author	Model type	Variables	Variable readily available?	Validation (I/E)	Accuracy	Metric	Sample size (events)	Outcome	Tx
**Zaorsky** [65]	Survival model	Score derived from: Age, PSA, Gleason Score, ADT, Radiation dose, Stages.	Yes	External validation of AJCC version 6	0.54	c-index	2469 (NR)	OS	3D-CRT, IMRT
**Zaorsky** [65]	Survival model	Score derived from: Age, PSA, Gleason Score, ADT, Radiation dose, Stages.	Yes	External validation of AJCC version 7	0.54	c-index	2469 (NR)	OS	3D-CRT, IMRT
**Vainshtein** [18]	Survival model (Cox)	CAPRA scores (based on PSA, Biopsy Gleason, Age at diagnosis, clinical tumour stage and % biopsy cores positive for cancer)	Yes	External validation of CAPRA	0.56	c-index	85 (NR)	PCSM	EBRT with long term Androgen deprivation
**Zaorsky** [65]	Survival model	Score derived from: Age, PSA, Gleason Score, ADT, Radiation dose, Stages.	Yes	External validation of AJCC version 7	0.58	c-index	2469 (NR)	OS	3D-CRT, IMRT
**Zaorsky** [65]	Survival model	Score derived from: Age, PSA, Gleason Score, ADT, Radiation dose, Stages.	Yes	External validation of AJCC version 6	0.52	c-index	2469 (NR)	BCF	3D-CRT, IMRT
**Zaorsky** [65]	Survival model	Score derived from: Age, PSA, Gleason Score, ADT, Radiation dose, tages.	Yes	External validation of AJCC version 7	0.6	c-index	2469 (NR)	BCF	3D-CRT, IMRT
**Vance** [60]	Survival model (Cox)	PSA, Gleason, clinical T stage, PCV, ADT use	Yes	Not stated	0.61 95% CI 0.53-0.68	c-index	599 (NR)	OS	EBRT
**Buyyounouski** [38]	Survival model	Interval to Biochemical failure (dicotomized at 18 months)	Yes	External validation of IBF	0.61; 95% CI 0.58-0.65; 48.4%; 86.1%	c-index; sensitivity; specificity.	1722 (290)	PCSM	EBRT
**Westphalen** [62]	Survival (Cox, Nomogram presented)	PSA level, clinical stage (from digital rectal examination findings), sum of Gleason grades, use of neoadjuvant ADT, and radiation dose	Yes	External validation of Kattan with additions	0.61; 95% CI 0.581-0.640	c-index	99 (30)	BCF	EBRT
**Qian** [52]	Survival model (Cox)	NCCN risk stratification tool plus percent positive cores	Yes	Not stated	0.63	c-index	652 (NR)	BFFF	3D-CRT, IMRT
**Vance** [60]	Survival model (Cox)	PSA, Gleason, clinical T stage, PCV, ADT use	No (prostate cancer volume)	Not stated	0.64; 95% CI 0.57-0.70	c-index	599 (NR)	BFFF	EBRT
**Qian** [52]	Survival model (Cox)	NCCN risk stratification tool plus percent positive cores	Yes	Not stated	0.64	c-index	652 (NR)	Metastases	3D-CRT, IMRT
**Vainshtein** [18]	Survival model (Cox)	CAPRA scores (based on PSA, Biopsy Gleason, Age at diagnosis, clinical tumour stage and % biopsy cores positive for cancer)	Yes	External validation of CAPRA	0.67	c-index	85 (NR)	BFFF	EBRT with long term Androgen deprivation
**Zelefsky** [66]	Survival (Cox, Nomogram presented)	ADT, T stage, Gleason, Pre PSA, RT dose.	Yes	Not stated	0.67	c-index	2551	BFFF	3D-CRT, IMRT
**Vance** [60]	Survival model (Cox)	PSA, Gleason, clinical T stage, PCV, ADT use	No (prostate cancer volume)	Not stated	0.67; 95% CI 0.60-0.74	c-index	599 (NR)	FFM	EBRT
**Zaorsky** [65]	Survival model	Score derived from: Age, PSA, Gleason Score, ADT, Radiation dose, Stages.	Yes	External validation of AJCC version 6	0.68	c-index	2469 (NR)	PCSM	3D-CRT, IMRT
**Halverson** [46]	Survival model (Cox)	CAPRA: PSA, T stage, Gleason score, percent positive biopsy, and age	Yes	External validation of CAPRA	0.69	c-index	612 (NR)	BFFF	EBRT
**Zaorsky** [65]	Survival model	Score derived from: Age, PSA, Gleason Score, ADT, Radiation dose, Stages.	Yes	External validation of AJCC version 6	0.70	c-index	2469 (NR)	DM	3D-CRT, IMRT
**Qian** [52]	Survival model (Cox)	NCCN risk stratification tool plus percent positive cores	Yes	Not stated	0.71	c-index	652 (NR)	PCSM	3D-CRT, IMRT
**Zelefsky** [68]	Survival (Cox, Nomogram presented)	T stage, Gleason Score, radiation dose, Neoadjuvant ADT, Pre-treatment PSA level,	Yes	Internal (bootstrapping)	0.72	c-index	2253 (578)	BCF	3D-CRT, IMRT
**Williams** [17]	Survival (Cox, Nomogram presented)	Age, prostate-specific antigen value, Gleason score, clinical stage, androgen deprivation duration, and radiotherapy dose	Yes	Not stated	0.72	c-index	3264 (1048)	BCF	EBRT
**Vainshtein** [18]	Survival model (Cox)	CAPRA scores (based on PSA, Biopsy Gleason, Age at diagnosis, clinical tumour stage and % biopsy cores positive for cancer)	Yes	External validation of CAPRA	0.73	c-index	153 (NR)	PCSM	EBRT with short term Androgen deprivation
**Steigler** [56]	Survival Model (Fine and Gray)	PSA doubling time (PSADT definition specified), time to biochemical failure, high risk category defined by PSADT <4 months or TTBF < 1 year and low risk category by PSADT >9 months or TTBF > 3 years.	Yes	Internal (bootstrapping)	0.73	c-index	485 (150)	PCSM	EBRT
**Vance** [60]	Survival model (Cox)	PSA, Gleason, clinical T stage, PCV, ADT use	No (prostate cancer volume)	Not stated	0.75; 95% CI 0.67-0.83	c-index	599 (NR)	PCSM	EBRT
**Zaorsky** [65]	Survival model	Score derived from: Age, PSA, Gleason Score, ADT, Radiation dose, Stages.	Yes	External validation of AJCC version 7	0.75	c-index	2469 (NR)	DM	3D-CRT, IMRT
**Sanpaolo** [21]	Survival (Cox, Nomogram presented)	Age, Gleason score, tumor stage, initial PSA, androgen deprivation therapy, pelvic radiotherapy, administered doses, days of radiotherapy, and biologically effective dose	Yes	Internal (bootstrapping)	0.75	c-index	670 (70)	BCF	3D-CRT
**Vainshtein** [18]	Survival model (Cox)	CAPRA scores (based on PSA, Biopsy Gleason, Age at diagnosis, clinical tumour stage and % biopsy cores positive for cancer)	Yes	External validation of CAPRA	0.78	c-index	612 (51)	FFM	EBRT
**Vainshtein** [18]	Survival model (Cox)	CAPRA scores (based on PSA, Biopsy Gleason, Age at diagnosis, clinical tumour stage and % biopsy cores positive for cancer)	Yes	External validation of CAPRA	0.79	c-index	374 (NR)	FFM	EBRT (no ADT)
**Vainshtein** [18]	Survival model (Cox)	CAPRA scores (based on PSA, Biopsy Gleason, Age at diagnosis, clinical tumour stage and % biopsy cores positive for cancer)	Yes	External validation of CAPRA	0.80	c-index	612 (23)	PCSM	EBRT
**Vainshtein** [18]	Survival model (Cox)	CAPRA scores (based on PSA, Biopsy Gleason, Age at diagnosis, clinical tumour stage and % biopsy cores positive for cancer)	Yes	External validation of CAPRA	0.80	c-index	153 (NR)	FFM	EBRT with short term Androgen deprivation
**Zaorsky** [65]	Survival model	Score derived from: Age, PSA, Gleason Score, ADT, Radiation dose, Stages.	Yes	External validation of AJCC version 7	0.81	c-index	2469 (NR)	PCSM	3D-CRT, IMRT
**Proust-Lima** [51]	Joint Model (Latent Class)	Repeat PSA measures	No	External (two separate cohorts *n* =503 and 615)	0.82	Weighted average error of prediction (WAEP) at 1 year; after 3 years 0.0614, 0.0095.	1268 (190)	Clinical recurrence	EBRT
**Vainshtein** [18]	Risk stratification	CAPRA scores (based on PSA, Biopsy Gleason, Age at diagnosis, clinical tumour stage and % biopsy cores positive for cancer)	Yes	External validation of CAPRA	0.86	c-index	374 (NR)	PCSM	EBRT (no ADT)
**Yu** [63]	Joint modelling	T stage, ln(PSA), Gleason, Age, dose, duration of RT, PSA, slope, HT, Baseline hazards, measurementerrors and tuning parameters.	No, baseline hazards, measurement errors, tuning parameters included	External (prospective on 612 patients from the original cohort)	Not stated	NA	928 (24)	BCF	EBRT
**Yu** [64]	Survival model (Cox)	Peri-neurial invasion, clinical T stage, Gleason, pre-treatment PSA, radiation dose, ADT	Yes	Not stated	Not stated	NA	657 (145)	BCF	EBRT
**Cooperberg** [40]	Survival model (Weibull parametric)	CAPRA scores (based on PSA, Biopsy Gleason, Age at diagnosis, clinical tumour stage and % biopsy cores positive for cancer)	Yes	External	Not stated	NA	1143 (NR)	10 year PCSM	EBRT
**Cooperberg** [39]	Survival model (Cox)	CAPRA scores (based on PSA, Biopsy Gleason, Age at diagnosis, clinical tumour stage and % biopsy cores positive for cancer)	Yes	External	Not stated	NA	1262 (62)	PCSM	EBRT
**Zumsteg** [69]	Survival model (Cox)	Stratification for NCCN intermediate risk patients based on: Gleason, % Positive biospy cores and number of intermediate risk factors	Yes	Not stated	Not stated	NA	424 (NR)	BFFF	EBRT
**Zumsteg** [69]	Survival Model (Fine and Gray)	Stratification for NCCN intermediate risk patients based on: Gleason, % Positive biospy cores and number of intermediate risk factors	Yes	Not stated	Not stated	NA	424 (NR)	PCSM	EBRT
**Zumsteg** [69]	Survival model (Cox)	Stratification for NCCN intermediate risk patients based on: Gleason, % Positive biospy cores and number of intermediate risk factors	Yes	Not stated	Not stated	NA	424 (NR)	LF	EBRT
**Zumsteg** [69]	Survival model (Cox)	Stratification for NCCN intermediate risk patients based on: Gleason, % Positive biospy cores and number of intermediate risk factors	Yes	Not stated	Not stated	NA	424 (NR)	DM	EBRT
**Zelefsky** [67]	Survival Model (Fine and Gray)	T stage, Gleason, RT dose, pre-RT PSA, Nadir PSA	Yes	Not stated	Not stated	NA	812 (81)	DM	3D-CRT, IMRT
**Zelefsky** [67]	Survival Model (Fine and Gray)	T stage, Gleason, RT dose, pre-RT PSA, Nadir PSA	Yes	Not stated	Not stated	NA	843 (65)	PCSM	3D-CRT, IMRT
**Zelefsky** [67]	Survival model (Cox)	T stage, Gleason, RT dose, pre-RT PSA, Nadir PSA	Yes	Not stated	Not stated	NA	769 (246)	BFFF	3D-CRT, IMRT
**Thames** [59]	Survival model (Cox)	T stage, Gleason Score, ln(initial PSA), PSA indicator interval, non-treatment day ratio, dose, Overall treatment time	No, Institution adjustment and PSA interval are cohort specific	Not stated	Not stated	NA	3426 (1445)	BCF	2D or 3D-CRT
**Taylor** [58]	Joint model (longitudinal and survival)	Gleason score, T stage, PSA before treatment, Dose and date of radiation, Serial PSA values after treatment	Yes	External (separate cohort not stated)	Not stated	NA	3232 (458)	Clinical recurrence (local, regional or distant)	EBRT
**Murgic** [50]	Survival model (Cox)	Age, PSA, T-stage, Gleason, ADT use, Pelvic RT, RT dose, Maximum biopsy core, percent positive cores	No, pelvic RT included	Not stated	Not stated	NA	590 (NR)	BFFF	EBRT
**Murgic** [50]	Survival model (Cox)	Age, PSA, T-stage, Gleason, ADT use, Pelvic RT, RT dose, Maximum biopsy core, percent positive cores	No, pelvic RT included	Not stated	Not stated	NA	590 (NR)	FFM	EBRT
**Murgic** [50]	Survival model (Cox)	Age, PSA, T-stage, Gleason, ADT use, Pelvic RT, RT dose, Maximum biopsy core, percent positive cores	Yes, pelvic RT included	Not stated	Not stated	NA	590 (NR)	PCSM	EBRT
**Murgic** [50]	Survival model (Cox)	Age, PSA, T-stage, Gleason, ADT use, Pelvic RT, RT dose, Maximum biopsy core, percent positive cores	Yes, pelvic RT included	Not stated	Not stated	NA	590 (NR)	OS	EBRT
**Spratt** [55]	Survival model (Cox)	Age, T-stage, Gleason score, pre-treatment PSA, >50% core involvement, use of ADT, and PSA density	Yes, PSA density can be calculated	Not stated	Not stated	NA	1002 (NR)	BCF	IMRT
**Spratt** [55]	Survival model (Cox)	Age, T-stage, Gleason score, pre-treatment PSA, >50% core involvement, use of ADT, and PSA density	Yes, PSA density can be calculated	Not stated	Not stated	NA	1002 (NR)	DMFS	IMRT
**Spratt** [55]	Survival Model (Fine and Gray)	Age, T-stage, Gleason score, pre-treatment PSA, >50% core involvement, use of ADT, and PSA density	Yes, PSA density can be calculated	Not stated	Not stated	NA	1002 (NR)	PCSM	IMRT
**Sabolch** [53]	Survival model (Cox)	Pre-treatment PSA, T-stage, Gleason score, GP5, ADT, and Charlson comorbidity index.	No, includes Charlson comorbidity index	Not stated	Not stated	NA	718 (NR)	BFFF	3D CT or IMRT
**Sabolch** [53]	Survival model (Cox)	Pre-treatment PSA, T-stage, Gleason score, GP5, ADT, and Charlson comorbidity index.	No, includes Charlson comorbidity index	Not stated	Not stated	NA	718 (NR)	Freedom from Metastases	3D CT or IMRT
**Sabolch** [53]	Survival model (Cox)	Pre-treatment PSA, T-stage, Gleason score, GP5, ADT, and Charlson comorbidity index.	No, includes Charlson comorbidity index	Not stated	Not stated	NA	718 (NR)	PCSM	3D CT or IMRT
**Sabolch** [53]	Survival model (Cox)	Pre-treatment PSA, T-stage, Gleason score, GP5, ADT, and Charlson comorbidity index.	No, includes Charlson comorbidity index	Not stated	Not stated	NA	718 (NR)	OS	3D CT or IMRT
**Huang** [47]	Survival model (Cox)	Gleason score, iPSA, and % positive cores	Yes	Not stated	Not stated	NA	1056 (176)	BCF	EBRT
**Huang** [47]	Survival Model (Fine and Gray)	Gleason score, iPSA, and % positive cores	Yes	Not stated	Not stated	NA	1056 (30)	PCSM	EBRT
**Huang** [47]	Survival model (Cox)	Gleason score, iPSA, and % positive cores	Yes	Not stated	Not stated	NA	1056 (634)	OS	EBRT
**Feng** [28]	Survival model (Cox); also recursive partitioning	age, race, T stage, PSA, No of biopsy cores taken, percent positive cores, Gleason Score, NCCN risk group, RT dose, Pelvic RT, ADT	No, includes pelvic RT	Not stated	Not stated	NA	651 (NR)	FFM	EBRT
**Feng** [28]	Survival model (Cox); also recursive partitioning	age, race, T stage, PSA, No of biopsy cores taken, percent positive cores, Gleason Score, NCCN risk group, RT dose, Pelvic RT, ADT	No, includes pelvic RT	Not stated	Not stated	NA	651 (NR)	PCSM	EBRT
**Feng** [28]	Survival model (Cox); also recursive partitioning	age, race, T stage, PSA, No of biopsy cores taken, percent positive cores, Gleason Score, NCCN risk group, RT dose, Pelvic RT, ADT	No, Includes pelvic RT	Not stated	Not stated	NA	651 (NR)	BFFF	EBRT
**Feng** [28]	Survival model (Cox); also recursive partitioning	age, race, T stage, PSA, No of biopsy cores taken, percent positive cores, Gleason Score, NCCN risk group, RT dose, Pelvic RT, ADT	No, includes Pelvic RT	Not stated	Not stated	NA	651 (NR)	OS	EBRT
**Engineer** [9]	Survival model (Cox)	Age, Tumour stage, Gleason score, PSA, ADT, radiation dose, period of treatment	No, includes period of treatment	Not stated	Not stated	NA	174 (21)	BFFF	2D or 3D-CRT
**Engineer** [9]	Survival model (Cox)	Age, Tumour stage, Gleason score, PSA, ADT, radiation dose, period of treatment	No, includes period of treatment	Not stated	Not stated	NA	174 (98)	Disease free survival	2D or 3D-CRT
**Engineer** [9]	Survival model (Cox)	Age, Tumour stage, Gleason score, PSA, ADT, radiation dose, period of treatment	No, includes period of treatment	Not stated	Not stated	NA	174 (124)	OS	2D or 3D-CRT
**Denham** [44]	Survival model (Cox)	Time to biochemical failure	Yes	Not stated	Not stated	NA	802 (125)	PCSM	EBRT
**Denham** [44]	Survival model (Cox)	PSA doubling time	No, multiple PSA measures required	Not stated	Not stated	NA	802 (125)	PCSM	EBRT
**D’Amico** [43]	Survival Model (Fine and Gray)	PSA velocity, biopsy Gleason score, PSA, and clinical stage	No, PSA velocity	Not stated	Not stated	NA	288 (32)	PCSM	3D-CRT
**Slater** [54]	Survival model (Cox)	NCCN grouping, percent positive biopsy cores (PPBC), percentage of cancer volume (PCV), maximum involvement of biopsy scores (MIBC)	No, percentage cancer volume	Not stated	Not stated	NA	398 (NR)	bNED	Proton and photonbeam therapy
**D’Ambrosio** [41]	Survival model (Cox)	Non-treatment day ratio, absolute number of non-treatment days, Gleason, pre-treatment PSA, T stage, radiation dose	No, includes treatment days	Not stated	Not stated	NA	1796 (NR)	BCF	3D-CRT, IMRT

*Abbreviations*: *OS* overall survival, *RT* radiotherapy, *BCF* bio chemical failure, *BFFF* bio chemical freedom from failure, *PCSM* prostate cancer specific mortality, *LF* local failure, *DM* distant metastases, *DMFS* distant metastases-free survival, *FFM* freedom from metastases, *TTBF* time to bio chemical failure, *STI* secondary therapeutic intervention, *bNED* bio chemical no evidence of disease, *2D-CRT* 2D - Conformal radiotherapy; *3D-CRT* 3D -Conformal radiotherapy, *EBRT* external beam radiotherapy, *NA* not applicable, *NR* not reported

**Table 4:** Prognostic tools relating to combinations of brachytherapy and external beam radiation therapy

Author	Model type	Variables	Variable readily available?	Validation (I/E)	Accuracy	Metric	Sample size (number of events)	Outcome	Tx
**Rodrigues** [14]	Survival model (Cox)	T stage, PSA and Gleason	Yes	Internal (cross validation)	0.64	c-index	7839 (NR)	OS	Brachytherapy and or EBRT
**Rodrigues** [14]	Survival model (Cox)	T stage, PSA and Gleason	Yes	Internal (cross validation)	0.67	c-index	7839 (NR)	BFFF	Brachytherapy and or EBRT
**Delouya** [19]	Survival model (Cox)	CAPRA score (Age, PSA, Gleason score, T-stage, PPB)	Yes	External	0.69, 95%CI 55.0 to 83.8; 0.66, 95%CI 54.4 to 78.3; 0.68, 95%CI 58.5 to 77.2; 0.62 95%CI 53.2 to 70.7	c-index at 2, 3, 4, and 5 years	744 (47)	BFFF	Brachytherapy or EBRT
**Delouya** [19]	Survival model (Cox)	D’Amico classification (T-stage, PSA and Gleason)	Yes	External	59.1% - 61.6%; and 54.5% - 61.6%	3-5 year sensitivity and specificity	744 (47)	BFFF	Brachytherapy or EBRT
**Wattson** [61]	Survival Model (Fine and Gray)	Number of high-risk factors (prostate-specific antigen >20 ng/mL, biopsy Gleason score 8–10, or clinical stage T2c), adjusted for age, comorbidity, and the type of supplemental treatment	No, comorbidity	Not stated	Not stated	NA	2234 (57)	PCSM	EBRT and or Brachytherapy
**Kubicek** [48]	Survival model	Mid therapy PSA (<25% vs > =25%)	No, mid therapy PSA cohort specific	Not stated	Not stated	NA	717 (NR)	Disease free survival	Brachytherapy and EBRT
**Kubicek** [48]	Survival model	Mid therapy PSA (<25% vs > =25%)	No, mid therapy PSA cohort specific	Not stated	Not stated	NA	717 (NR)	OS	Brachytherapy and EBRT
**Krishnan** [20]	Survival model (Cox)	CAPRA scores (based on PSA, Biopsy Gleason, Age at diagnosis, clinical tumour stage and % biopsy cores positive for cancer)	Yes	External	Not stated	NA	345 (45)	BCF	EBRT and/or LDR
**McKenna** [49]	Survival model (Cox)	Patient age, hormonal treatment, baseline PSA, and degree of extracapsular extension, pre-treatment MRI	Yes, where MRI is routine	Not stated	Not stated	NA	80 (4)	Metastatic recurrence and BCF	EBRT or EBRT with Brachytherapy

*Abbreviations*: *OS* overall survival, *BCF* bio chemical failure, *BFFF* bio chemical freedom from failure, *PCSM* prostate cancer specific mortality, *NA* not applicable, *NR* not reported, *MRI* magnetic resonance imaging

The 47 papers finally included in this review described 97 individual predictive models. Of these models, 16 related to brachytherapy treatment (Table 2), 72 to external beam radiation therapy (Table 3) and nine to a combination of brachytherapy and external beam radiation therapy (Table 4).

Across all radiation treatment modalities, outcomes relating to PSA levels post treatment were most common (39 models) followed by prostate cancer specific mortality (29 models). Measures of metastases (17) and overall survival (14 models) were less common (note that some papers report more than one outcome and model). Of those studies reporting development of new models (66), only nine reported validation either internally or in an additional cohort. Only 67/97 (69%) models included variables which were considered to be readily available in existing data sets.

Critical appraisal considered the criteria set by the CEBM appraisal tool for prognostic studies [9]. Risk of bias ranged from moderate (Q1; Was the defined representative sample of patients assembled at a common point in the course of their disease? (72%), Q2; Was patient follow-up sufficiently long and complete? (64%)) to low (Q3; Were outcome criteria either objective or applied in a ‘blind’ fashion? (85%), Q4; If subgroups with different prognoses are identified, did adjustment for important prognostic factors take place? (91%)) (Table 5).

**Table 5:** Risk of bias assessment summary table

Study Id	Q1	Q2	Q3	Q4
Cooperberg [39]	high	low	low	low
Bittner [27]	high	low	high	low
Buyyounouski [38]	low	low	low	low
Cooperberg (41)	low	high	low	low
Delouya [19]	low	high	low	low
Engineer [9]	low	high	low	low
Feng [28]	low	low	low	low
Frank [25]	unclear	high	low	low
Frank [45]	unclear	low	unclear	low
Halverson [46]	low	low	low	low
Huang [47]	low	low	low	low
Kaplan [12]	unclear	high	low	low
Krishnan [20]	low	high	low	low
Kubicek [48]	low	low	low	high
Marshall [11]	unclear	low	low	low
Potters [16]	unclear	high	low	low
Rodrigues [14]	high	unclear	low	low
Proust-Lima [51]	low	low	unclear	low
Sabolch [53]	low	low	low	low
Sanpaolo [21]	low	low	low	low
Slater [54]	high	low	low	low
Spratt [55]	low	low	low	low
Steigler [56]	low	low	low	unclear
Taylor [58]	low	low	unclear	low
Vainshtein [18]	low	low	low	low
Vance [60]	low	low	low	low
Wattson [61]	low	high	low	low
Westphalen [62]	unclear	high	low	low
Williams [17]	low	high	low	low
Yoshida [15]	unclear	low	unclear	low
Zaorsky [65]	low	low	low	low
Zelefsky [10]	low	high	low	low
Zelefsky [68]	low	low	low	low
Zelefsky [66]	low	low	low	low
Zumsteg [69]	low	low	low	low
D’Amico [43]	low	high	low	low
Yu [64]	low	low	low	low
D’Ambrosio [41]	unclear	low	low	low
Denham [44]	low	unclear	low	low
McKenna [49]	unclear	high	low	high
Yu [63]	low	unclear	unclear	low
D’Amico [42]	low	low	low	low
Zelefsky [67]	low	low	low	low
Thames [59]	low	low	unclear	low
Qian [52]	low	low	low	low
Sylvester [57]	low	low	low	high
Murgic [50]	low	high	low	low
Low/47	34 (72%)	30 (64%)	40 (85%)	43 (91%)

Q1: Was the defined representative sample of patients assembled at a common (usually early) point in the course of their disease)? Q2: Was patient follow-up sufficiently long and complete? Q3: Were outcome criteria either objective or applied in a ‘blind’ fashion? Q4: If subgroups with different prognoses are identified, did adjustment for important prognostic factors take place?

High = high risk of bias, low = low risk of bias, unclear = unclear if study design is at high or low risk of bias

### Brachytherapy

In regards to models predicting outcomes following brachytherapy, Potters et al. [17] report the highest c-index in a model developed and internally validated using a cohort of 5,931 patients. This model predicts 9 year freedom from biochemical failure and remains to be validated externally. Eleven models relating to brachytherapy (69%) did not report model accuracy and among those models which did report accuracy, all related to biochemical failure endpoints. Three studies report to be external validations of the Prostogram nomogram (also known as the Kattan nomogram), all of which have low c-indices (0.49, 0.51 and 0.66) suggesting that this model is of limited clinical utility. A c-index of 1 ‘indicates a perfect ability to rank the outcomes in the order they actually occurred (100% sensitivity and specificity), whereas 0.5 is a purely random ranking and is analogous to the area under the receiver operator characteristic curve’ (definition from [18]).

The majority of papers identified in this review reported models relating to external beam radiation therapy (72/97 = 74%). Fifty-four percent (39 of 72) of these models did not have their accuracy reported. 61% of models did not report validation (either internal or external, including external validation of already published models).

### External beam radiation therapy

The model relating to external beam radiation therapy with the highest accuracy was described by Vainshtein [19], which was an external validation of the CAPRA stratification in the context of external beam radiation therapy. The cohort included 374 patients and the endpoint of prostate cancer specific mortality was predicted with c-index of 0.86. Accuracy of this model is also reported for the outcome of biochemical failure and subgroups of patients receiving long term ADT or short term ADT, all which had lower accuracy.

### External beam radiation therapy with brachy therapy

Nine models were identified which were specific to patients treated with external beam radiation therapy in combination with brachytherapy. Of these models, five (56%) did not report accuracy. The highest accuracy was reported by Delouya [15, 20] (c-index 0.69) predicting biochemical failure free survival at 2-years. This study was based on a cohort of 744 patients and was an external validation of the CAPRA score. Prediction at 5-years was achieved with c-index 0.62.

## Discussion

Since the publication of previous reviews, there has been considerable progress in the field of outcomes prediction following prostate cancer treatment. This review identified 47 papers published between 2007 and 2015, which describe 97 predictive tools for men receiving radiotherapy. This includes 66 models which were newly developed and 31 which were validations of already published predictive tools. Consistent with previous reports, most tools (65%) are yet to be validated in a population outside the derivation set. Studies were included from 2007 as the modality of radiation therapy has changed significantly over the past decade, and historic data may not be a useful basis for prognosis. Apart from modality, the total dose has also significantly increased however, we found that only five studies [13, 16, 20–22] did not use data from men treated as far back as the 1990s.

The volume of research carried out in the field of prognostics has exploded over the last decade. A systematic review that included all studies published before July 2007 (the cut-off date for inclusion in the present review) identified 17 studies on prognostic models that related to prostate cancer patients treated with radiotherapy [4]. In this review 39 new studies were identified which investigated prognostic markers for BCF. Unfortunately, the majority of new studies did not undertake validation, mirroring the finding of the previous systematic review. As validation – particularly external validation – is vital for the appropriate clinical implementation of prognostic models, this suggests that resources and efforts are not being efficiently targeted to improve tools available for clinical practice.

With regards to the methodological quality of the literature, our critical appraisal found that overall studies were at low to moderate risk of bias. The greatest risk was created by insufficient follow-up (defined as a mean or median of ≥5 years) which only occurred in 64% of studies. There was also a moderate risk of bias created by the possibility of included patients being at different points in the course of their prostate cancer, however in the majority of cases this was due to insufficient specificity in the description of inclusion criteria as opposed to reported differences. There was little risk of bias created by the measurement of outcomes, as the main outcomes (biochemical failure [various definitions], metastasis, survival) were objective, or by a lack of adjustment for important prognostic factors as the essential factors of prostate cancer prognosis (PSA, Gleason score, and clinical stage) were used nearly universally.

Model accuracy was not reported in 57% of the models included. Model accuracy was reported to be highest in Vainshtein 2014 [23] with a c-index of 0.86 derived for prediction of prostate cancer specific mortality with the CAPRA score (originally established in [24]), including the addition of variables for the presence of Gleason 5 and treatment with ADT (this c-index relates to patients not receiving ADT). This study acts to externally validate the CAPRA scoring system (with modifications) in patients treated with external beam radiation therapy, though this improvement to the score requires further validation in other populations. Of the remaining 42 models which reported predictive accuracy, c indices were typically in the 0.70–0.80 range which would be considered ‘reasonable’ according to Hosmer and Lemeshow [25]. Notably, those papers which did not report external validation typically had higher c-indices suggesting that original model developments should be considered optimistic in their predictive capacity. The lowest c-index (0.49, 95%CI 0.37 to 0.61) was reported for a study [26] performing external validation of the Prostogram nomogram (originally established in [27]) suggesting this nomogram may have little predictive value.

The predictive tools identified in this review included joint-modelling approaches but not neural networks which have featured in previous reviews. This may reflect a change in statistical tools available since publication of earlier catalogues [4]. Two of the survival models [28, 29] did not account for competing risks when predicting prostate cancer specific mortality, a potential weakness which could easily be addressed.

The majority of papers attempted prediction relating to biochemical recurrence, prostate cancer specific mortality or overall survival with a smaller subset predicting metastases. Sixteen of the 97 models identified related to brachytherapy with 72 for external beam radiation therapy and 9 a combination of the two. This could reflect more wide-spread use of external beam radiation therapy, and we might anticipate more tools relating to HDR brachytherapy (with or without EBRT) in the future. There is a dearth of externally validated nomograms focusing on brachytherapy and brachytherapy in combination with external beam radiation therapy particularly looking at overall survival and cancer specific survival outcomes.

This study did not explicitly set out to uncover tools incorporating novel variables, but only those which could be used in current clinical settings. Despite this, 31% of studies included reference to variables which have been less studied to date (e.g. mid-point PSA levels). While such variables may prove useful, there is currently limited opportunity to validate these observations using existing datasets. It is possible that additional variables including standardised measures of comorbidity, imaging features or genetic markers, which are becoming more accessible may help to improve the accuracy of future models. For a recent review of potential molecular and genetic candidate see Hall et al. 2016 [30].

Most predictive tools identified in this review were developed in US populations. This observation should be considered by clinicians who are based outside the US when selecting a predictive model to assist treatment decision making. Where possible, tools validated in a setting similar to one’s own clinical practice should be selected for use. The number of tools available internationally would be increased with additional validation work conducted outside the US and particularly in multi-national cohorts.

We observed a large degree of variation in the quality of reporting clinical predictive tools. This may stem from the fact that authors are not aware of reporting guidelines in the field or indeed that such guidelines exist. The TRIPOD guidelines (http://www.equator-network.org/reporting-guidelines/tripod-statement/) for reporting of multivariable prediction models were published in March 2015, shortly before the cut-off for papers included in this review. These guidelines have been widely endorsed and published in key journals [31–39]. Further publication of multivariable models would benefit greatly from adherence to these guidelines.

## Conclusions

Tools which aid decision making offer more accurate prediction of clinical outcomes when compared to clinical judgement alone. This understanding has led to a large increase in the number of predictive tools relating to clinical outcomes post radiation therapy between 2007 and 2015. This review identifies 47 papers describing 97 models published in the period, a substantial increase compared to the 17 models previously described between 1966 and 2007. Of the models identified, 65% had no external validation and 57% did not report accuracy. Thirty one percent of models included variables which are not part of typical registry data sets, and are therefore difficult to validate. Despite these limitations, there are accurate and externally validated models for external beam radiation therapy treatment which predict prostate cancer specific mortality. There are fewer models which accurately predict outcomes following brachytherapy (alone or in combination with external beam radiation therapy). This review provides an accessible catalogue of predictive tools which could be used currently (i.e. those with high accuracy after external validation) and identifies those which should be prioritised for future validation.

## Additional file

Additional file 1: Reasons for exclusion from the review. (DOCX 21 kb)

## Abbreviations

BCR: Biochemical recurrence
BF: Biochemical failure
EBRT: External beam radiation therapy
OS: Overall survival
PCSM: Prostate cancer specific mortality
TTP: Time to progression
