# Supplementary material for: An appraisal of analytical tools used in predicting clinical outcomes following radiation therapy treatment of men with prostate cancer: a systematic review
Source: Radiat Oncol. 2017 Mar 21;12:56. doi: 10.1186/s13014-017-0786-z (PMC5359887; doi:10.1186/s13014-017-0786-z)
Supplement: Additional file 1: — Reasons for exclusion from the review. (DOCX 21 kb) [file 13014_2017_786_MOESM1_ESM.docx]

## Additional file 1: Table S1a Reasons for exclusions

| Reference | Reason for exclusion |
| --- | --- |
| Alcantara, P.; Hanlon, A.; Buyyounouski, M. K.; Horwitz, E. M.; Pollack, A. *Prostate-specific antigen nadir within 12 months of prostate cancer radiotherapy predicts metastasis and death.* Cancer Jan 1 2007;109(1):41-7 | Outside criteria dates < July 2007 |
| Bowes D.; Crook JM.; Wallace K.; Evans A.; Toi A.; Finelli A.; Jewett MA.; Fleshner N.; Catton C. *Results of a surgically derived nomogram to predict Gleason score upgrading applied to a cohort of patients with "favorable-risk" prostate cancer treated with permanent seed brachytherapy.* Urology Sep 2012;80(3):649-55 | Not prognostic |
| Budäus, Lars; Graefen, Markus; Salomon, Georg; Isbarn, Hendrik; Lughezzani, Giovanni; Sun, Maxine; Chun, Felix K. H.; Schlomm, Thorsten; Steuber, Thomas; Haese, Alexander; Koellermann, Jens; Sauter, Guido; Fisch, Margit; Heinzer, Hans; Huland, Hartwig; Karakiewicz, Pierre I. *The novel nomogram of Gleason sum upgrade: Possible application for the eligible criteria of low dose rate brachytherapy.* International Journal of Urology 2010;17(10):862-868 | Relates to RP not RT |
| Buyyounouski, Mark K.; Hanlon, Alexandra L.; Horwitz, Eric M.; Pollack, Alan. *Interval to biochemical failure highly prognostic for distant metastasis and prostate cancer-specific mortality after radiotherapy.* Jan 1 2008;70(1):59-66 | Outside criteria dates < July 2007 |
| Crook, Juanita M.; Malone, Shawn; Perry, Gad; Eapen, Libni; Owen, Julie; Robertson, Susan; Ludgate, Charles; Fung, Sharon; Lockwood, Gina. *Twenty-four-month postradiation prostate biopsies are strongly predictive of 7-year disease-free survival: results from a Canadian randomized trial.* Feb 1 2009;115(3):673-9 | Secondary analysis of RCT |
| Denham, James W.; Steigler, Allison; Wilcox, Chantelle; Lamb, David S.; Joseph, David; Atkinson, Chris; Tai, Keen-Hun; Spry, Nigel A.; Gleeson, Paul S.; D'Este, Catherine. *Why are pretreatment prostate-specific antigen levels and biochemical recurrence poor predictors of prostate cancer survival?* Oct 1 2009;115(19):4477-87 | Secondary analysis of RCT |
| Hamstra, D. A.; Bae, K.; Pilepich, M. V.; Hanks, G. E.; Grignon, D. J.; McGowan, D. G.; Roach, M.; Lawton, C.; Lee, R. J.; Sandler, H. *Older Age Predicts Decreased Metastasis and Prostate Cancer-Specific Death for Men Treated with Radiation Therapy: Meta-Analysis of Radiation Therapy Oncology Group Trials.* International Journal of Radiation Oncology Biology Physics Dec 2011;81():1293-1301 | Secondary analysis of RCT |
| Katz, Matthew S.; Efstathiou, Jason A.; Damico, Anthony V.; Kattan, Michael W.; Sanda, Martin G.; Nguyen, Paul L.; Smith, Matthew R.; Carroll, Peter R.; Zietman, Anthony L. *The CaP Calculator: an online decision support tool for clinically localized prostate cancer.* BJU BJU International 2010;105(10):1417-1422 | Description of online tool (to calculate predictions from multiple nomograms simultaneously). |
| Liauw, Stanley L.; Liauw, Sun H. *Prolongation of total treatment time because of infrequently missed days of treatment is not associated with inferior biochemical outcome after dose-escalated radiation therapy for prostate cancer.* Nov 1 2011;81(3):751-7 | Not prognostic |
| Maffezzini, M.; Bossi, A.; Collette, L. *Implications of prostate-specific antigen doubling time as indicator of failure after surgery or radiation therapy for prostate cancer.* Eur Urol Mar 2007;51(3):605-13; discussion 613 | Review |
| Maffezzini, M.; Bossi, A.; Collette, L. *Implications of prostate-specific antigen doubling time as indicator of failure after surgery or radiation therapy for prostate cancer.* Eur Urol Mar 2007;51(3):605-13; discussion 613 | Outside criteria dates < July 2007 |
| Nanda, A.; Chen, M. H.; Renshaw, A. A.; D'Amico, A. V. *Gleason Pattern 5 Prostate Cancer: Further Stratification of Patients With High-Risk Disease and Implications for Future Randomized Trials.* 2009;74(5):1419-1423 | Cohort includes RP |
| Nguyen, P. L.; Chen, M. H.; Catalona, W. J.; Moul, J. W.; Sun, L.; D'Amico, A. V. *Predicting Prostate Cancer Mortality among Men with Intermediate to High-Risk Disease and Multiple Unfavorable Risk Factors.* International Journal of Radiation Oncology Biology Physics Mar 2009;73():659-664 | Cohort includes RP |
| Papagikos, M. A.; Rossi, P. J.; Urbanic, J. J.; deGuzman, A. F.; McCullough, D. L.; Clark, P. E.; Lee, W. R. *A simple model predicts freedom from biochemical recurrence after low-dose rate prostate brachytherapy alone.* Am J Clin Oncol Apr 2007;30(2):199-204 | Outside criteria dates < July 2007 |
| Pe, Mark L.; Trabulsi, Edouard J.; Kedika, Ramalinga; Pequignot, Edward; Dicker, Adam P.; Gomella, Leonard G.; Valicenti, Richard K. Effect of percentage of positive prostate biopsy cores on biochemical outcome in low-risk PCa treated with brachytherapy or 3D-CRT. Jun 2009;73(6):1328-34 | Not prognostic |
| Potters, L.; Morgenstern, C.; Calugaru, E.; Fearn, P.; Jassal, A.; Presser, J.; Mullen, E. *12-Year Outcomes Following Permanent Prostate Brachytherapy in Patients with Clinically Localized Prostate Cancer.* Journal of Urology 2008;179(5 SUPPL.):S20-S24 | Outside criteria dates < July 2007 |
| Proust-Lima, C.; Taylor, J. M.; Williams, S. G.; Ankerst, D. P.; Liu, N.; Kestin, L. L.; Bae, K.; Sandler, H. M. *Determinants of change in prostate-specific antigen over time and its association with recurrence after external beam radiation therapy for prostate cancer in five large cohorts.* Int J Radiat Oncol Biol Phys Nov 1 2008;72(3):782-91 | Outcome not a standard clinical measure |
| Qian, Yushen; Feng, Felix Y.; Halverson, Schuyler; Blas, Kevin; Sandler, Howard M.; Hamstra, Daniel A. *The percent of positive biopsy cores improves prediction of prostate cancer-specific death in patients treated with dose-escalated radiotherapy.* Nov 1 2011;81(3):e135-42 | Duplicate |
| Spalding, A. C.; Daignault, S.; Sandler, H. M.; Shah, R. B.; Pan, C. C.; Ray, M. E. *Percent positive biopsy cores as a prognostic factor for prostate cancer treated with external beam radiation.* Urology May 2007;69(5):936-40 | Outside criteria dates < July 2007 |
| Stock, Richard G.; Klein, Thomas J.; Cesaretti, Jamie A.; Stone, Nelson N. *Prognostic significance of 5-year PSA value for predicting prostate cancer recurrence after brachytherapy alone and combined with hormonal therapy and/or external beam radiotherapy.* Jul 1 2009;74(3):753-8 | Comparative effectiveness |
| Walz J; Gallina A; Saad F; Montorsi F; Perrotte P; Shariat, S.; Jeldres C; Graefen M; Benard F; McCormack M; Valiquette, L.; Karakiewicz, P. I. *A Nomogram Predicting 10-Year Life Expectancy in Candidates for Radical Prostatectomy or Radiotherapy for Prostate Cancer.* Journal of Clinical Oncology 2007;25(24):3576-3581 | Cohort includes RP |
| Walz, Jochen; Gallina, Andrea; Hutterer, Georg; Perrotte, Paul; Shariat, Shahrokh F.; Graefen, Markus; McCormack, Michael; Benard, Francois; Valiquette, Luc; Saad, Fred; Karakiewicz, Pierre I. *Accuracy of Life Tables in Predicting Overall Survival in Candidates for Radiotherapy for Prostate Cancer.* International Journal of Radiation Oncology Biology Physics 2007;69(1):88-94 | Outside criteria dates < July 2007 |
| Westphalen, A. C.; Koff, W. J.; Coakley, F. V.; Muglia, V. F.; Neuhaus, J. M.; Marcus, R. T.; Kurhanewicz, J.; Smith-Bindman, R. *Prostate cancer: prediction of biochemical failure after external-beam radiation therapy--Kattan nomogram and endorectal MR imaging estimation of tumor volume.* Radiology Nov 2011;261(2):477-86 | Duplicate |
| Yamamoto, T.; Ito, K.; Miyakubo, M.; Takechi, H.; Suzuki, K.; Akimoto, T.; Ishikawa, H.; Nakano, T. *Nomogram ranking as new objective evaluation method in various treatment strategies for patients with prostate cancer with various clinicopathologic backgrounds.* Urology Oct 2008;72():892-897 | Not prognostic |
| Zakeri K.; Rose BS.; Gulaya S.; D'Amico AV.; Mell LK. *Competing event risk stratification may improve the design and efficiency of clinical trials: secondary analysis of SWOG 8794.* Contemporary clinical trials Jan 2013;34(1):74-9 | Secondary analysis of RCT |

## Additional file 1: Table S1b Reasons for exclusions (summary)

| Reason for Exclusion | Number of Papers |
| --- | --- |
| Dates | 5 |
| Duplicate | 2 |
| Not prognostic, secondary analysis of RCT or comparative effectiveness | 9 |
| Review | 2 |
| RP | 4 |
| Other | 3 |
| Grand Total | **25** |
